# Effect of Thermal and Non-Thermal Technologies on Kinetics and the Main Quality Parameters of Red Bell Pepper Dried with Convective and Microwave–Convective Methods

**DOI:** 10.3390/molecules27072164

**Published:** 2022-03-27

**Authors:** Katarzyna Rybak, Artur Wiktor, Mohammad Kaveh, Magdalena Dadan, Dorota Witrowa-Rajchert, Małgorzata Nowacka

**Affiliations:** 1 Department of Food Engineering and Process Management, Institute of Food Sciences, Warsaw University of Life Sciences—SGGW, 02-787 Warsaw, Poland; katarzyna_rybak@sggw.edu.pl (K.R.); artur_wiktor@sggw.edu.pl (A.W.); magdalena_dadan@sggw.edu.pl (M.D.); dorota_witrowa_rajchert@sggw.edu.pl (D.W.-R.); 2 Department of Petroleum Engineering, College of Engineering, Knowledge University, Erbil 44001, Iraq; sirwan.kaweh@knu.edu.iq

**Keywords:** ultrasound, pulsed electric field, blanching, drying, porosity, color, polyphenols, carotenoids, sugars content, antioxidant activity

## Abstract

The drying process preserves the surplus of perishable food. However, to obtain a good-quality final product, different pretreatments are conducted before drying. Thus, the aim of the study was the evaluation of the effect of thermal (blanching treatments with hot water) and non-thermal technologies (pulsed electric field (PEF) and ultrasound (US)) on the kinetics of the drying process of red bell pepper. The convective and microwave–convective drying were compared based on quality parameters, such as physical (water activity, porosity, rehydration rate, and color) and chemical properties (total phenolic content, total carotenoids content, antioxidant activity, and total sugars content). The results showed that all of the investigated methods reduced drying time. However, the most effective was blanching, followed by PEF and US treatment, regardless of the drying technique. Non-thermal methods allowed for better preservation of bioactive compounds, such as vitamin C in the range of 8.2% to 22.5% or total carotenoid content in the range of 0.4% to 48%, in comparison to untreated dried material. Moreover, PEF-treated red bell peppers exhibited superior antioxidant activity (higher of about 15.2–30.8%) when compared to untreated dried samples, whereas sonication decreased the free radical scavenging potential by ca. 10%. In most cases, the pretreatment influenced the physical properties, such as porosity, color, or rehydration properties. Samples subjected to PEF and US treatment and dried by using a microwave-assisted method exhibited a significantly higher porosity of 2–4 folds in comparison to untreated material; this result was also confirmed by visual inspection of microtomography scans. Among tested methods, blanched samples had the most similar optical properties to untreated materials; however non-thermally treated bell peppers exhibited the highest saturation of the color.

## 1. Introduction

Red bell peppers are rich in nutrients and antioxidants and are used around the world for flavoring or medicinal purposes [1]. Due to the high amount of water (more than 75%), red bell pepper has the potential to germinate, rot, and ultimately reduce its shelf life [2], causing many economic losses to the sellers. Therefore, to increase shelf life and maintain nutritional value, a suitable processing method should be used to reduce the moisture content of the red bell pepper [3].

The drying process can remove ninety percent of the water in the food, thereby reducing the microorganism’s growth and food spoilage and the negative effect of moisture on food properties, as well as reducing transportation costs. However, some undesirable physical and chemical changes, such as discoloration, texture, and nutritional value, may also occur during drying, which reduces consumer acceptance [4]. At present, the methods of agricultural products’ drying are mainly divided into traditional and unconventional methods [5,6]. Traditional methods are based on thermal drying, including hot-air-drying. At present, in many developing countries, traditional methods are used to dry agricultural products, and this reduces the quality and increases the waste. Traditional drying methods lead to increased shrinkage of the tissue with a hard surface, adverse changes in color, aroma, taste, and reduced rehydration rate, and nutritional value of the product. Furthermore, hot-air-drying consumes a lot of energy due to low efficiency of the devices [7]. Therefore, different heat sources are used combined with hot-air-drying, such as infrared radiation or microwaves. Foodstuff dried by using the microwave method is often characterized by better quality (lesser shrinkage or higher content of bioactive components), due to the shorter drying time, by around 95% [8], compared to hot-air-drying, or around 65% for intermittent microwave-drying [9]. What is more, the cost of the process is also significantly reduced. Thus, choosing the right method for drying or using the appropriate pretreatments before the drying process can greatly improve the quality characteristics of the final dried product. Pretreatment also has very important advantages, such as preventing discoloration of the fruit by inactivating the enzyme, reducing the drying time by loosening the fruit texture, and increasing the quality of the fruit after drying [10]. Different pretreatment methods are applied before drying, such as blanching or dipping in different solutions [11]. Furthermore, non-thermal methods can also be used to increase mass and heat transfer processes, including ultrasound (US) and pulsed electric field (PEF).

Being subjected to US before drying may lead to an increase in the amount of moisture leaving the food during the drying process, including the increase in temperature in the boundary layer, pressure change due to cavitation, the development of microchannels as a result of shear stress cavitation, and perturbation in the boundary layer [12]. The PEF pretreatment, which has a different mechanism than the US, involves the use of short pulses with high electric field intensities to treat food between two electrodes. Cellular biological systems after PEF pretreatment may undergo electroporation, which gives reversible or irreversible effects. Due to the formation and growth of membrane pores, the permeability of the membrane increases [13]. There are many examples of successful applications of PEF and US in food processing, taking into account kinetics, as well as physical and chemical properties of the final product [14,15].

Thermal treatment as blanching is an important unit operation that can be applied before the industrial drying of fruits and vegetables to inactivate enzymes, enhance process kinetics, and prevent some quality degradation [16]. It is well-known that blanching also causes undesirable changes, especially those associated with textural and nutrients changes [17]. However, non-thermal treatment, such as US and PEF, allow us to achieve similar technological aims through different mechanisms of action—and also because low temperatures for treatment positively affect the plant tissue. Thus, in this study, the effect of thermal (blanching treatments with hot water) and non-thermal technologies (PEF and US) on the kinetics of convective and microwave–convective drying were investigated. Furthermore, dried red bell peppers obtained with two different methods were compared based on quality parameters, such as physical (water activity, porosity, rehydration rate, and color) and chemical properties (total phenolic content, total carotenoids content, antioxidant activity, and total sugars content). Such an extensive approach comparing drying methods and applied thermal and non-thermal treatments, as well as physical and chemical properties of red bell pepper, has not been studied comprehensively before.

## 2. Results and Discussion

### 2.1. Convective and Microwave–Convective Drying Kinetics

**Figure 1:**
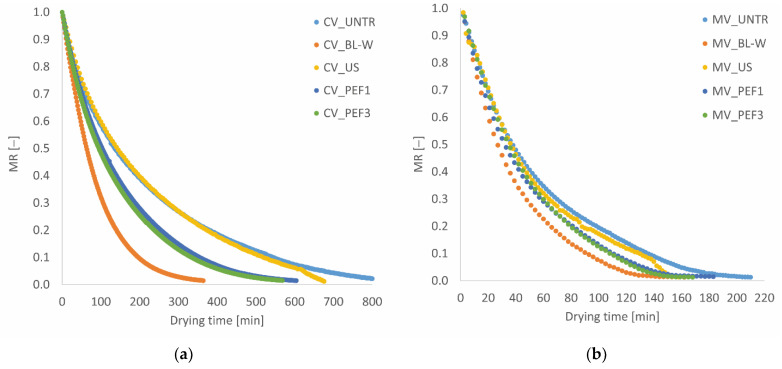
Drying kinetics for (**a**) convective (CV) and (**b**) microwave–convective (MV) dried red bell pepper without and with subjected thermal or non-thermal treatment prior to drying.

The data obtained in convective and microwave–convective dryers and various pretreatments (blanching in water, ultrasound, and pulsed electric field) for drying red bell pepper are shown in Figure 1. Our examination of Figure 1a,b shows that, in the initial stages of drying, the descent slope of drying speed is high, and in later stages, this slope decreases. This can be due to the high amount of moisture released in the early stages of the drying process. Over time, the moisture content of the product decreases, followed by a decrease in the rate of moisture released from the product, and the drying speed of the red bell pepper layers decreases, followed by an increase in the drying time. This is observed in the final stages of the drying process [18]. Furthermore, it can be seen that, in the microwave dryer, as compared to the convective dryer, the drying time is reduced. Using the microwave power increases the slope of the line of the moisture ratio chart, thus reducing the drying time. On the other hand, the use of microwave power increases the moisture-absorption capacity of the air, due to the increase in temperature difference between the air and the product, and causes the product to heat up faster and water to evaporate better. As a result, the drying time is reduced [19,20]. Furthermore, the use of higher power for the microwave reduced the drying time. The difference in kinetics of air-drying and microwave-assisted drying is associated with the different mechanisms of water evaporation. More intense water removal in the case of microwave-assisted drying is caused by the volumetric heating of the sample due to absorption of electromagnetic field and rapid water dipoles movement [21].

When comparing the two types of dryers with different pretreatments, the results showed that the maximum drying time (810 min) was obtained in the convective dryer for the control sample (Table 1). The use of pretreatment compared to the control mode, regardless of the methods of drying, reduces the drying time. In pretreated samples, as compared to the control one, the change in moisture transfer rate from the center of the sample to the surface increases [22]. Moreover, the density and integrity of the tissue are reduced, due to the use of different pretreatments compared to the control mode, and moisture is removed more quickly from the layers of red bell pepper. On the other hand, by examining the effect of different pretreatments in different dryers, it can be seen that blanching pretreatment can reduce drying time more than the other two pretreatments (US and PEF). This may be because blanching pretreatment inactivates enzymes, removes oxygen from intercellular spaces, and results in faster mass transfer in these samples [16,23]. In our earlier studies [24], when blanching conducted in water was used as a pretreatment method before the freeze-drying process, the drying time of red bell pepper decreased around 72%, and this is associated with the damage of internal structures in the tissue [25,26].

Sonication, as well as pulsed electric field, results in microstructure changes, which facilitate the water removal during drying. Sonication may results in the formation of microchannels that can serve as a path for water migration, whereas PEF ruptures cellular membrane integrity, which is the main limiting factor of mass transfer during the drying of plant-origin materials [27,28]. In comparison to untreated dried samples, the application of non-thermal technologies significantly shortened the convective drying process by 17.8%, 31.6%, and 35.1% for US, PEF1, and PEF3, respectively. A similar trend was obtained for microwave–convective drying; however, in this case, decreased dehydration time in comparison to the control operation was not statistically significant for samples treated with US and PEF.

### 2.2. Quality Parameters of Red Bell Pepper Dried with Convective and Microwave–Convective Methods

#### 2.2.1. Physical Properties of Dried Red Bell Pepper

##### Water Activity, Porosity, and Rehydration Rate of Convective and Microwave–Convective Dried Red Bell Pepper

Water activity is a very important parameter which influences the growth of microorganisms, as well as many biochemical processes, in plant tissue. The value of water activity below 0.6 protects food against the growth of microorganisms, and the dried materials can be stored for a long time [29]. Dried red bell pepper obtained water activity in the range of 0.201 to 0.314 in a convective dryer and from 0.242 to 0.393 in a microwave dryer, and this is much below the value of 0.6. The dried materials to which different pretreatment methods (BL-W, US, and PEF) were applied were characterized by significantly lower water activity in comparison to the untreated dried samples (Table 2). The use of PEF3 treatment in the CV dryer and US treatment in the MV dryer, with 0.201 and 0.242, had the lowest amount of water activity, respectively.

**Figure 2:**
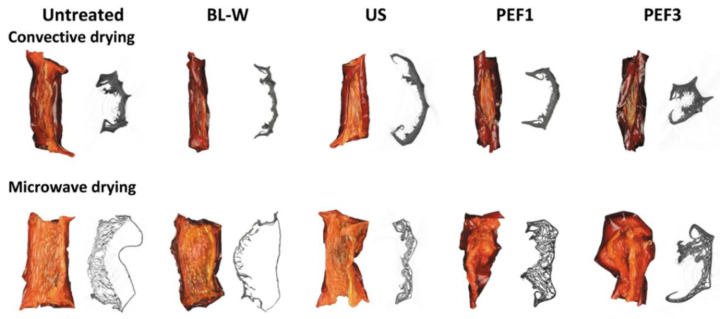
Photos (in color) and binarized μCT images (black-and-white color) of convective (CV) and microwave–convective (MV) dried red bell pepper without (untreated) and subjected to thermal or non-thermal treatment prior to drying.

The drying process and evaporating water from the plant material result in texture, as well as porosity, changes [30]. Similarly, the pretreatments applied prior to the drying process have effects on the rupturing cell membranes and change the porosity of the cell wall [26]. The porosity of the dried red bell pepper was in the range of 4.7% to 16.4% for convective drying, and for the microwave–convective method, the drying porosity was higher, in the range of 8.5% to 39.9%. The higher porosity of samples processed by microwave-assisted drying is associated with the volumetric heating and evaporation of water in the samples. The traditional pretreatment (BL-W) results in higher or similar porosity in comparison to the untreated dried sample. Moreover, Ciurzyńska et al. [26], after blanching processing, as well as for a sonicated sample, obtained lower shrinkage and higher porosity of freeze-dried red beets compared to a sample without pretreatment. In our study, red bell pepper subjected to US and PEF pretreatment also improved porosity; however, the drying method had a more significant impact in this case. Dried materials with the use of microwaves and pretreated with non-thermal technologies prior to drying were characterized as having a significantly higher porosity than those dried with the convective method. This has also been confirmed by microtomography (XRCT) examinations (Figure 2). The XRCT images showed (to facilitate observation raw images were inverted in black and white colors) that, during the microwave-drying of red bell pepper, the puffing effect occurs. A similar effect was observed by other scientists when microwaves were used [31,32]. On the other hand, convective drying caused significant material shrinkage, and this is in line with previous studies [33].

The structure and porosity have an influence on the rehydration properties. According to Table 2, the final rate of the RR of convective and microwave–convective methods and various pretreatments is statistically significant. The highest RR was related to MV dryer and US pretreatment, i.e., 4.68. Meanwhile, in the CV dryer, the highest amount of RR was related to BL-W (4.21). The use of US can enlarge the capillaries and weaken the internal structure of the product by cavitation and mechanical effects [34,35], and this phenomenon is useful for improving the RR of the sample. Moreover, in the case of PEF treatment, a higher RR was also noticed; however, the specific energy intake (3 kJ/kg) resulted in a similar lower value of RR. Fauster et al. [36] noticed that freeze-dried strawberries tissue, as well as bell pepper, was characterized by reduced shrinkage, due to cell disintegration, which positively influences the rehydration capacity. On the other hand, Shynkaryk et al. [37] showed that the PEF treatment may negatively affect the texture, and the rehydration capacity is lower than that for untreated freeze-dried red beetroots.

##### Color of Convective and Microwave–Convective Dried Red Bell Pepper

**Figure 3:**
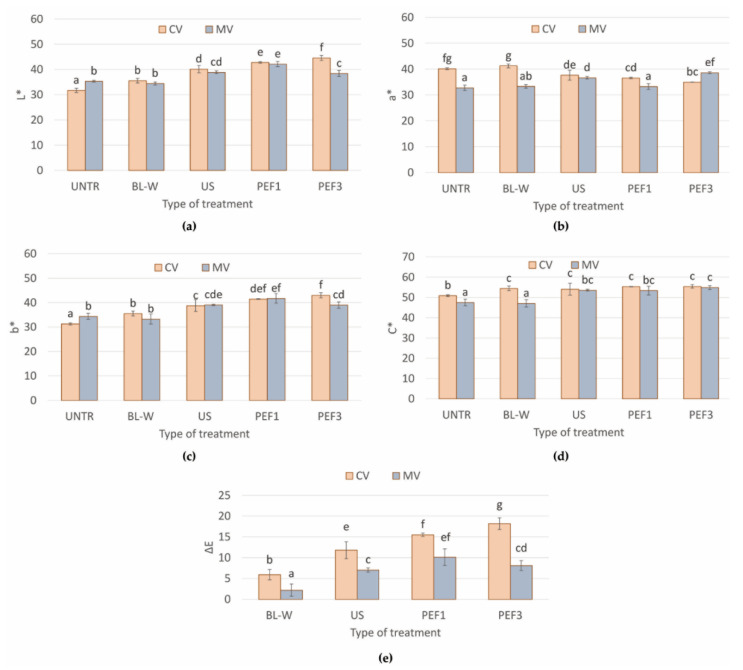
Color parameters: (**a**) L*—lightness, (**b**) a*—share of red color, (**c**) b*—share of yellow color, (**d**) C*—saturation, and (**e**) ∆E—total color difference (in comparison to untreated dried sample) of convective (CV) and microwave–convective (MV) dried red bell pepper subjected to thermal or non-thermal treatment prior to drying. Column presents a mean (from at least 6 repetitions), and errors bars show standard deviation. The different letters above columns show different homogeneous groups (Tukey’s HSD, α = 0.05).

The relevant results of the effect of the drying process and pretreatment methods on changes of L*, a*, and b* color parameters and total color difference (∆E) are summarized in Figure 3. The changes of the color parameters were dependent on the type of applied treatment and methods of drying. Generally, convective drying of red pepper, non-thermal-treated prior to drying, causes darkening of tissue; increases L*, b*, and C*; and lowers a* parameter in comparison to untreated CV dried sample (Figure 3a–d). Similar alterations were observed for material dried with microwave–convective drying; however, the changes were smaller. This might be related to the shorter drying time of microwave-drying (Figure 1) in comparison to the convective process. Moreover, microwaves might cause higher inactivation of enzymatic activity responsible for the darkening of the material. However, this theory explains further research for final confirmation. Moreover, the applied microwave power had an influence on color parameters [21].

In all of these methods, the color of the samples changes over time during drying; however, in some methods, the intensity of the changes is very high. The best-case scenario is when the rate of total color difference (∆E) is very low. The type of drying and pretreatment method used for drying strongly affects the plant tissue color. According to Figure 3e, the least changes of ∆E are related to the sample dried in the microwave dryer and subjected before drying to the blanching pretreatment. When hot air was applied in convective drying, slightly higher changes were observed (Figure 3e). However, still, the ∆E was lower in comparison to the samples treated with the non-thermal process. This is linked with the blanching process, which prevents the enzyme’s reaction [38]. Thus, the color changes in the blanched material during drying do not occur so fast as in the tissue, where some enzymes are still active after US and PEF pretreatment [39,40]. The value of ΔE also may be attributed to the drying time (Table 1), because the shorter drying time minimizes color degradation and retains the color properties of dried bell peppers.

#### 2.2.2. Chemical Properties of Dried Red Bell Pepper

##### Total Polyphenol Content, Total Carotenoid Content, and Antioxidant Activity (DPPH and ABTS Assay) of Convective and Microwave–Convective Dried Red Bell Pepper

**Figure 4:**
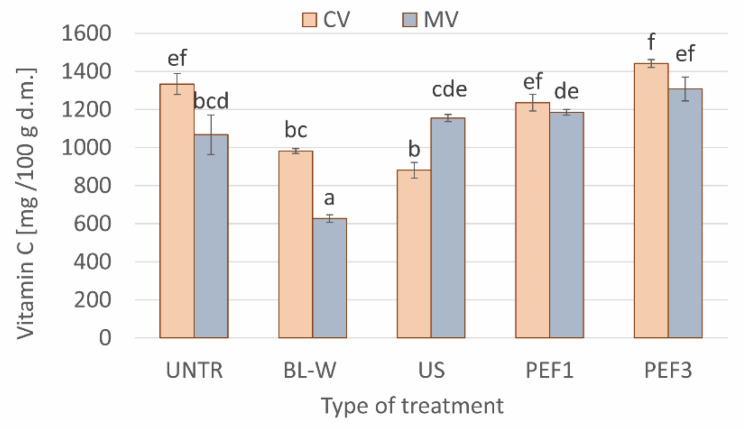
Comparison of vitamin C content for convective (CV) and microwave–convective (MV) dried red bell pepper subjected to thermal or non-thermal treatment prior to drying. Column presents a mean (from 3 repetitions), and errors bars show standard deviation. The different letters above columns show different homogeneous groups (Tukey’s HSD, α = 0.05).

Vitamin C is very sensitive to high temperatures, and the assessment of the amount of this compound can be used as an indicator of the overall quality of food [41]. With increasing temperature and time of the process, the content of vitamin C decreases in plant materials [42]. Thus, it was expected that blanching reduces the content of vitamin C (Figure 4). Surprisingly, convective drying, despite the fact that it lasted longer, allowed us to retain a higher content of vitamin C compared to drying with the use of microwaves. It is known that microwaves do not heat the product uniformly, and, therefore, the product may overheat or even burn [9]. This probably happened in the microwave–convective-dried red bell pepper, and it led to greater losses of vitamin C.

The application of the non-thermal processes before drying resulted in an unchanged or significantly lower amount of vitamin C in red bell pepper when compared to intact dried samples. These changes are the result of many processes taking place during the non-thermal pretreatment, such as stimulation by PEF and US treatment of the stress reaction of the plant tissue; better extraction of bioactive compounds, due to microstructure alterations caused by cavitation and sponge effect (US) [43]; or electroporation (PEF) [44]. On the other hand, during the US and PEF application, the reactive oxygen species (ROS) can be formed [45]; moreover, both processes are also conducted in water, meaning that water-soluble vitamin C [46] may be easily lost during the pretreatment.

**Figure 5:**
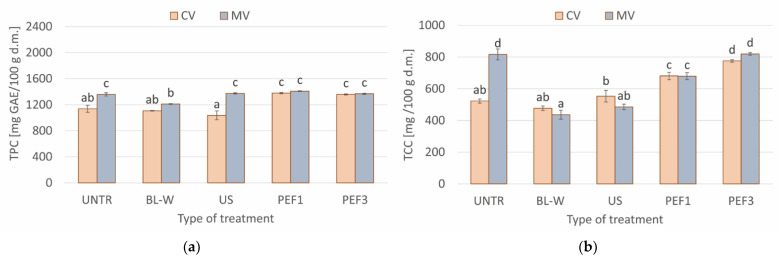
Comparison of (**a**) total phenolic content (TPC) and (**b**) total carotenoids content (TCC) for convective (CV) and microwave–convective (MV) dried red bell pepper subjected to thermal or non-thermal treatment prior to drying. Column presents a mean (from 3 repetitions), and errors bars show standard deviation. The different letters above columns show different homogeneous groups (Tukey’s HSD, α = 0.05).

The total phenolic content (TPC) and total carotenoids content (TCC) in dried red bell pepper subjected to different thermal and non-thermal treatments are presented in Figure 5a,b, respectively. The bioactive components contents were dependent upon both the pretreatment type and the drying method. The use of the convective method of red bell pepper drying resulted in a TPC in a range of 1037–1379 mg GAE/100 g d.m. (dry matter), whereas, for the microwave–convective method, it was 1212–1409 mg GAE/100 g d.m. The microwave-dried material was therefore characterized by a significantly higher (UNTR, US) or statistically unchanged content of polyphenols in comparison to the commonly utilized convective method. In turn, the TCC was analogous in CV- and MV-dried red bell pepper, except for untreated material, for which the microwaves contributed to obtaining a higher content of carotenoids. Probably the shorter drying time in the case of the microwave method was the reason for such a tendency. Analogous conclusions were made by Arslan and Özcan [47], who analyzed the antioxidant ability of red bell pepper dried by convective and microwave methods.

What is interesting is that the thermal treatment, which was blanching in water, contributed to degradation of polyphenols when microwave-drying was used, but in convective-dried red bell pepper, the TPC was statistically the same as in untreated material. In turn, the non-thermal treatments—US or PEF—resulted in a higher TPC in comparison to blanched red bell pepper when considering the MV method of drying, but statistically intact compared with the untreated sample. Perhaps a high temperature caused the degradation of polyphenols. On the other hand, for the CV drying method, the PEF significantly increased the TPC in comparison to both untreated and BL-W samples. For instance, the samples treated by PEF followed by CV drying were characterized by 19–21% higher TPC in comparison to untreated CV dried red bell pepper. There were no differences between TPC in both PEF samples, irrespective of the further drying method. In our previous study [48], the TPC in untreated juice from red bell pepper was equal to 2984 mg GAE/100 g d.m. When the tissue was subjected to PEF 1 kJ/kg, the statistically irrelevant difference was noted (2994 mg GAE/100 g d.m.), but when 3 kJ/kg was applied during PEF, the TPC was significantly lower (2824 mg GAE/100 g d.m.) than in untreated material. A higher TPC in the PEF-treated sample in current study was probably related to preservation of thermolabile compounds, due to a shortening of drying time by this non-thermal treatment, and/or a higher extractivity, due to the electroporation phenomenon, as it was reported previously in the literature [49].

As in the case of TPC, a similar tendency was observed in the case of TCC in CV red bell pepper. Both blanching and US applied before convective drying did not change the carotenoids contents in comparison to untreated material. Despite a high difference in the drying time, there were no differences in TCC between red bell pepper subjected to blanching or US, irrespective of the drying method. In other words, the shortest drying time for blanched samples prevented neither the polyphenols nor carotenoids. However, PEF increased their content by 30 (PEF1) and 48% (PEF3) due to a higher extraction ability caused by the electroporation phenomenon. The higher the specific energy intake during PEF treatment, the higher was the TCC. It seems that the US was not as effective at enhancing the bioactive components. The microwave-drying resulted in a different tendency of TCC in red bell pepper than it was after the convective method. Significantly, the highest TCC in MV-dried samples was noted for the untreated and PEF3 treated samples. Other treatments contributed to a higher degradation of carotenoids in MV-dried red bell pepper. Probably a higher specific energy intake during PEF caused a higher extraction of these components from the material. In the case of blanched and sonicated materials dried by the MV method, the degradation of carotenoids could occur because of the high temperature [50] or free radicals’ formation [51], respectively. In these samples, the benefits regarding the shorter drying time were not observed. In a previous study, it was proven that too high of a dose of energy delivered to the sample (treatment + microwave energy during drying) contributed to greater degradation of bioactive components [52].

**Figure 6:**
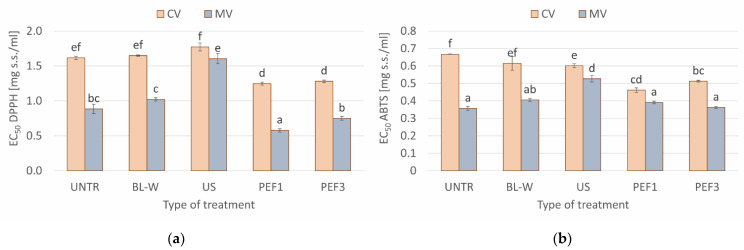
Comparison of antioxidant activity for convective (CV) and microwave–convective (MV) dried red bell pepper subjected to thermal or non-thermal treatment prior to drying, expressed as the effective concentration of the sample (EC_50_), which can decrease radicals by 50%: (**a**) 1,1-diphenyl-2-picrylhydrazyl radical (DPPH) and (**b**) 2,2-azinobis (3-ethylbenzothiazoline-6-sulfonate) radical cation (ABTS). Column presents a mean (from 3 repetitions), and errors bars show standard deviation. The different letters above columns show different homogeneous groups (Tukey’s HSD, α = 0.05).

The antioxidant activity of dried red bell pepper, as measured by DPPH and ABTS assay, is presented in Figure 6a,b, respectively. Lower values of EC50 indicate higher free radical scavenging potential. In general, the tendencies obtained by DPPH- and ABTS-based methods were alike. However, regardless of the variant of the experiment, the ABST method showed lower values of EC50. Such results are in accordance with our previously published data [24]. Among all evaluated samples, the materials dried by microwave-assisted air-drying (MV) exhibited better antioxidant activity in comparison to convective-dried material, no matter the pretreatment step. Such a situation was most probably related to the shorter drying time and, hence, shorter exposition to elevated temperature and oxygen presence. This explanation is supported by the results of Pearson’s correlation analysis—the relationship between EC50 and drying time was significant, and it was equal to r = 0.635 and r = 0.748 for DPPH and ABTS assays, respectively. A better performance of microwave-assisted air-drying than traditional convection technique in the conservation of antioxidant activity was also reported for jamun pulp [53], kiwi, and pepino fruits [54]. Among the studied samples, PEF-pretreated bell peppers exhibited the highest antioxidant activity, whereas ultrasound treatment led to the lowest free radical scavenging activity. Such results are closely related to the mechanism of those treatments and their technological execution. Both methods, PEF and US, can cause a rupture in the cellular structure and leakage of intracellular content, including antioxidants. Such a process makes them more available for many reactions that explain lower EC_50_ values of PEF treated bell peppers. However, the US treatment lasted much longer than the PEF application, and antioxidants could leak out of the material into an aqueous medium. Another explanation can be related to the fact that both techniques can lead to free radical and reactive oxygen species formation or their better penetration across ruptured cell membranes [55,56,57], and this may decrease the antioxidant activity. Once again, a longer application time in the case of the US compared to the PEF seems to play an important role. It is worth emphasizing that PEF1 conditions resulted in better antioxidant activity, as measured by DPPH assay, even compared to untreated samples. The ABTS method was less sensitive for the changes.

**Figure 7:**
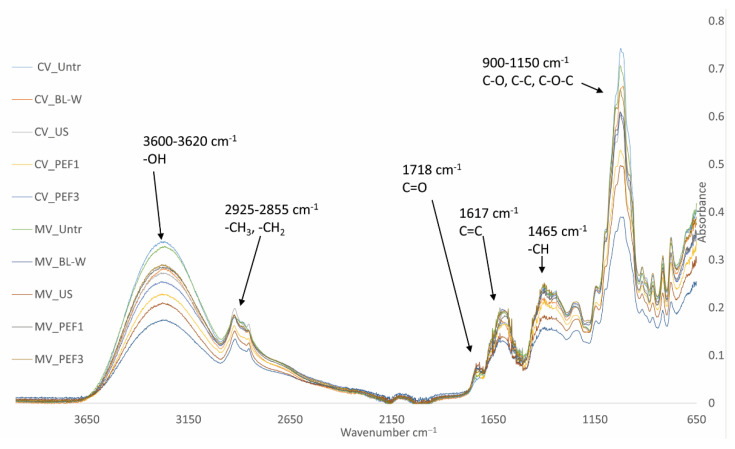
Fourier-transform infrared (FTIR) spectra of convective (CV) and microwave–convective (MV) dried red bell pepper subjected to thermal or non-thermal treatment prior to drying.

The FTIR spectra obtained for the dried red bell pepper contain functional groups present in molecules of compounds characteristic of plant materials, such as phenolic compounds, carotenoids, sugars, and vitamins (Figure 7). The infrared bands were assigned based on previous studies [58]. In the 3600–3620 cm^−1^ region, a strong band of stretching vibrations of the hydroxyl group coming from water and phenolic compounds is visible. The bands around 2925 and 2855 cm^−1^ correspond to the asymmetric stretching and deformations of the -CH_2_ and -CH_3_ alkyl groups. The samples have bands at 1718 cm^−1^ that correspond to the vibrational vibrations of the carbonyl group (C=O). The stretching vibrations of the C=C bonds occurring in carotenoids are 1617 cm^−1^. There were strong stretching vibrations for the groups: (C-O) and (C-C) groups were recorded in the area of 900–1150 cm^−1^, which is for different saccharides groups, such as glucose, fructose, and sucrose [59]. The dried materials’ spectra revealed differences in the absorbance intensities of the main functional groups that play a key role in the antioxidant properties of red bell peppers. The highest decrease is visible for the material subjected to heat treatment and dried by using the microwave–convection method (MV_BL-W).

##### Total Sugars Content of Convective and Microwave–Convective Dried Red Bell Pepper

**Figure 8:**
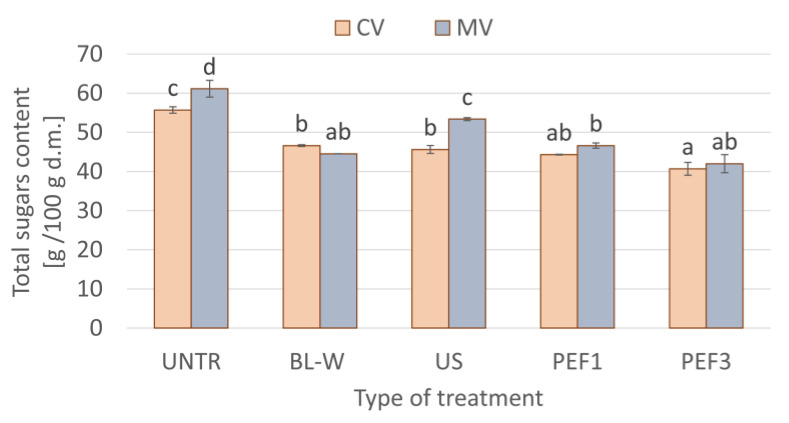
Comparison of total sugars content for convective (CV) and microwave–convective (MV) dried red bell pepper subjected to thermal or non-thermal treatment prior to drying. Column presents a mean (from 3 repetitions), and errors bars show standard deviation. The different letters above columns show different homogeneous groups (Tukey’s HSD, α = 0.05).

The total sugar content for convective (CV) and microwave–convective (MV) dried red bell pepper subjected prior to drying to thermal or non-thermal treatment is presented in Figure 8. Fresh red bell pepper contains sugars around 63.1 g/100 g d.m. [45], and dried samples were characterized by 55.7 ± 0.8 and 61.1 ± 2.1 g/100 g d.m. sugars content, respectively, for CV and MV. For all samples subjected to the pretreatment, the significantly lower total sugars content of 12.7 to 31.3% was noted. The initial processing, both thermal and non-thermal, causes injury to plant tissue and cell-wall rupturing, and, due to carrying out the process in water, this leads to the dissolution of sugars. The heating during blanching causes the disruption of the cells and the dissolving of the sugars, as well as other components soluble in water, such as organic acids, amino acids, minerals, etc. [60]. In addition, during ultrasound treatment, which is conducted in a water medium, the structural damages [61] result in leaking compounds soluble in water to the surroundings, especially when the sonication is conducted for a longer time [62]. Similarly, in the case of the PEF treatment, the electroporation causes a rupture in the cell membrane, and leaking of sugars is observed [63]. Soleto et al. [64] noted that, after PEF treatment of the red cherries, the sugars were released to the surface of the material. Thus, in most cases, in our studies, there were no significant differences in the sugar content between the studied samples.

**Figure 9:**
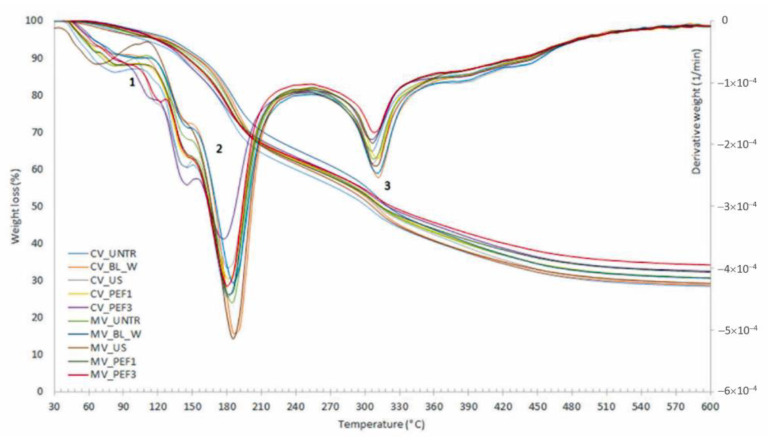
Phases of thermal degradation analysis (TGA and DTG) of convective (CV) and microwave–convective (MV) dried red bell pepper subjected to thermal or non-thermal treatment prior to drying.

Figure 9 shows the temperatures and weight losses depending on the phase of thermal degradation (TGA) for red bell peppers subjected to convective and microwave–convective drying and/or thermal (BL-W) or non-thermal (US and PEF) pretreatment. All dried samples showed three phases of thermal decomposition. In phase I (30–110 °C), mass losses result from evaporation and loss of water, as well as losses of volatile substances. The dried material contained a low amount of water; thus, the mass losses were not large, i.e., in the range of 3.1–5.2%. In phase II, in the temperature range from 110 to 240 °C, the highest weight losses in the range of 31.8–35% of the tested dried materials were observed. In this phase, thermal degradation of glucose and sucrose occurs at temperatures about 150–200 °C and 170–212 °C, respectively [65]. In the third phase, the temperature range was from 240 to 380 °C, while phase IV was from 380 to 600 °C. In the third phase, there was a high loss of mass in the range of 20.2–24.0%, which could result from the degradation of organic compounds. Then, in the last phase, the mass losses were similar for each of the dried samples and ranged from 9.4% to 10.6%. Polysaccharides, such as hemicellulose, are degraded at temperatures from about 220 to 315 °C, where cellulose was from about 250 to 450 °C and lignin from about 320 to 450 °C or even up to 1000 °C [66,67]. Thus, it can be summarized that mono- and disaccharides were decomposed in phase II, while in phase III, the initial degradation of polysaccharides starts, and in phase IV, it continues, where also lignocellulosic components were decomposed.

#### 2.2.3. Cluster Analysis

**Figure 10:**
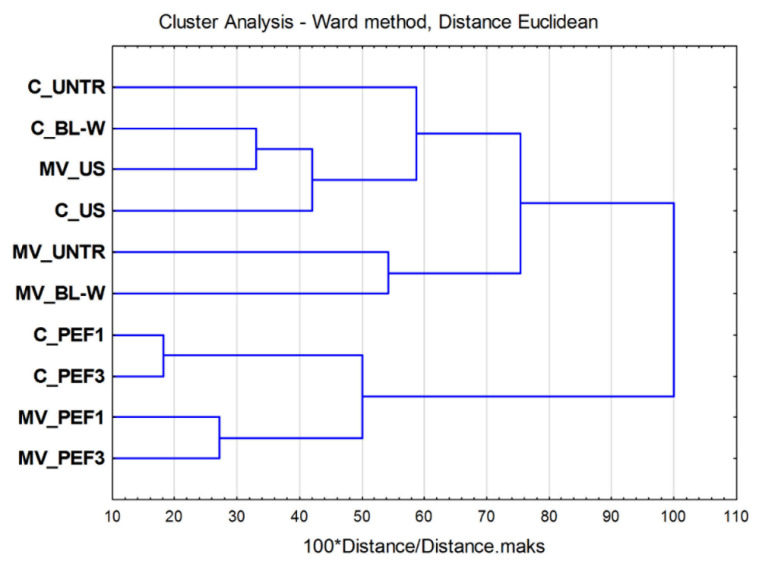
Results of Cluster Analysis of obtained results (parameters taken into account: TPC, TCC, vitamin C, DPPH, ABTS, sugars, L*, a*, b*, C*, ∆E, water activity, porosity, rehydration ratio, and drying time). C—convective drying, MV—microwave-assisted convective drying, UNTR—untreated, BL-W—blanched, US—ultrasound, PEF1—pulsed electric field treatment at 1 kJ/kg, PEF3—pulsed electric field treatment at 3 kJ/kg.

A complex statistical analysis of the obtained results was performed by using hierarchical cluster analysis, taking into account the following parameters: drying time, water activity, porosity, rehydration rate, color parameters (L*, a*, b*, C*, and ∆E), total polyphenols content, total carotenoids content, vitamin C content, antioxidant activity (DPPH^•^ and ABTS^•+^), and total sugars content. Its results are presented in Figure 10.

Two major groups were distinguished. In the first group, both samples dried by convection and microwave-assisted convection technique were agglomerated. A deeper analysis of this cluster showed that two subgroups formed according to the drying method. It is worth emphasizing that the bell peppers dried by convective method exhibited less dissimilarity than samples dried by the microwave-assisted technique. Such data may indicate that the application of more energy (3 kJ/kg) does not result in any added value benefits from a statistical point of view. Another cluster was bigger in the count and consisted of two subgroups at a dissimilarity level of ca. 75%. One of the subgroups was formed by untreated and blanched red bell peppers dried by the microwave-assisted method, whereas the second one consisted of material subjected to no treatment, blanching, or ultrasound application followed by convection drying or ultrasound treatment, followed by microwave-assisted air-drying.

## 3. Materials and Methods

### 3.1. Material

The fruits of the red pepper (*Capsicum annuum* L.) came from a local supplier (Bronisze, Poland). All drying operations were performed within 5 days of raw material purchase. The material was stored under monitored conditions (95% RH, 5 °C). Directly before the test, the material was washed up in cold tap water and gently dried with filter paper. The seeds and placenta were removed from the fruit, and the pericarp was cut into 2 × 4 cm pieces.

### 3.2. Experimental Design

The red bell pepper was subjected to pretreatments: blanching in water (BL-W), 30 min of sonication (US), and pulsed electric field with an energy input of 1 (PEF1) and 3 kJ/kg (PEF3). The parameters of the treatment were chosen on the basis of earlier experience and the preliminary experiments associated with the assessment of the structural changes by electrical properties measurements (unpublished data). In the case of US, 30 min of the treatment was selected as the treatment that increased electrical conductivity (43 µS/cm) significantly, but did not increase temperature more than 5 °C, whereas longer treatment led to significant increase of sample temperature. In the case of PEF low (PEF1) and high (PEF3), specific energy intake was studied as the protocol that led to two different levels of electrical conductivity (165 and 533 µS/cm, respectively). Application of higher specific energy input (>3 kJ/kg) was not related with any further noticeable increment of electrical conductivity (treatment at 5 kJ/kg was associated with electrical conductivity of 546 µS/cm). The pretreatment was performed in at least two repetitions. After pretreatment, samples were subjected to convective drying (CV) or microwave-drying (MV). The parameters of the sample treatment are shown in Table 3.

### 3.3. Thermal and Non-Thermal Treatment

#### 3.3.1. Thermal Treatment: Blanching (BL-W)

Sliced fruits were placed in hot tap water in a ratio of 1:2. The process was carried out at the temperature of 98 °C for 3 min, with stirring every 15 s. After treatment, the samples were filtered on a sieve, poured for 15 s with water at 15 °C, and gently dried with a paper towel to remove the residual water. The processing was performed in two replications.

#### 3.3.2. Non-Thermal Treatment: Ultrasound (US) and Pulsed Electric Field (PEF)

Ultrasound and pulsed electric field of various energies were used as alternative and innovative methods of pretreatment.

The immersion method was used for the sonication process (US). The cut material was placed directly in the laboratory chamber of an ultrasonic bath filled with water at a temperature in the range of 19.5–21.9 °C. The mass ratio of liquid to sample was 4:1. The sonotrodes located at the bottom of the device (ultrasonic bath MKD-3, MKD Ultrasonics, Warsaw, Poland) generated 300 W and a frequency of 21 kHz. The processing was performed two times.

The pulse electric field (PEF) treatment was performed by using an Elea PEF system (Elea Vertriebs- und Vermarktungsgesellschaft mbHVer, Quakenbrück, Germany), which delivers 2 Hz frequency of exponential decay pulses (monopolar signal, width of 40 ms). The red bell pepper (200 g of 2 × 4 cm pieces) was placed in the PEF system chamber with water of conductivity of 220 μS/cm and a temperature of 21 ± 1 °C. The electrodes were made from stainless steel, and the gap between them was 28 cm. The samples were treated with a specific energy consumption of 1 and 3 kJ/kg with of the same electric field intensity of 1.07 kV/cm. Technological experiments were conducted at least two times.

### 3.4. Drying

Microwave–convection drying was carried out in a Promis—Tech laboratory dryer (Wrocław, Poland) [68]. The material was placed as one layer perpendicular to the air flow on a rotating round sieve, receiving a load of 0.22 kg/m^2^. The process was conducted with the use of microwave power of 300 W, temperature of 40 °C, and air flow of 2 m/s. The mass and temperature of the sample were recorded continuously every 5 min. The microwave generator was turned off during the weighing process. Drying was finished to constant weight when the 3 consecutive weights did not change.

Convection drying was performed in a laboratory dryer (Warsaw, Poland), with continuous monitoring of the temperature of dried material and the mass. The air heated to the temperature of 50 °C flowed at the speed of 1.5 m/s parallel to the material layer. The load on the sieve was 1.45 kg/m^2^. The process was discontinued after reaching a stable mass.

Drying processes were performed in duplicates. In the samples after pretreatment and drying, the dry substance content was determined by using the gravimetric method at 105 °C for 24 h [69]. On the basis of the weight loss obtained during the drying processes of the red bell pepper tissue, drying curves were prepared as a function of the water ratio (MR) as a function of time, using the following equation [70]:

(1) $$MR=u_{\tau }/u_{0},$$

where *u*_0_ is the initial moisture content (kg H_2_O/kg dry matter (dm)), and *u*_τ_ is the moisture content at τ moment of the drying (kg H_2_O/kg dm).

The dried material was packed in three-layer bags made of aluminum composite film (12 μ PET/7 μ aluminum/100 μ PE) and sealed. The packages were stored at room temperature.

### 3.5. Quality of Dried Red Bell Pepper

#### 3.5.1. Physical Properties of Dried Red Bell Pepper

##### Water Activity of Dried Red Bell Pepper

The water activity in the product was measured 7 days after drying. The material was measured at 25 °C on a water activity mater with a dew point detector (4TE, AquaLab, Decagon Devices, Inc., Pullman, WA, USA). Three replications were performed.

##### Porosity of Dried Red Bell Pepper

The apparent geometric density (ϱ_s_) of the dried materials was calculated. The weighed material was placed in a glass class A laboratory cylinder and covered with sea sand to the cylinder volume of 25 mL. In order to determine the density (ρ_d_), taking into account also the pores closed in the sample, the weighed material was placed in the measuring cell of a helium stereopycnometer (SPY-6DC, Quantachrome Instruments, Boynton Beach, FL, USA) with a volume of 25 cm^3^. Before the measurement, the cell was rinsed 3 times with helium. The results were calculated by using the pycnometer software (version 2.7 from Quantachrome). Measurements were made in three replications. The obtained values of both densities were used to calculate the porosity of the material according to the following equation [24]:

P[%] = (1 − ϱ_d_/ϱ_s_) 100%, (2)

##### Rehydration Rate of Dried Red Bell Pepper

To measure the rehydration rate (RR), dried samples of red bell pepper were immersed in a beaker containing distilled water at 20 °C for three hours. After the desired time, the samples were taken out of the solutions and weighed after taking surface water. Then the water reabsorption ratio was calculated [71]:

(3) $$RR=R_{\tau }/Rd$$

where *RR* is the rehydration rate, *R*_τ_ is the sample weight after rehydration rate, and *Rd* is the dry sample weight.

##### Color of Dried Red Bell Pepper

Color was measured by using a colorimeter (Chroma Meter CR-5; Konica Minolta, Tokyo, Japan). The dried material was homogenized in an analytical mill (IKA A11 basic; IKA-Werke GmbH, Staufen im Breisgau, Germany) and placed in an optical glass Petri dish with the following dimensions: diameter of 45 mm and height of 17 mm. Standard light source was D65; observation angle: di: 8° and observer 2° were used to measure reflectance. The color was described in the CIE L*a*b* model. A white-and-black standard was used for calibration. On the basis of the basic components (L—brightness, a—share of green/red color, and b—share of blue/yellow color), basic color indexes were calculated: saturation C* and absolute color difference ∆E between the fresh material and the dried product [71]. Measurements were made in 6 replications.

##### Photos and X-ray—CT Images of Dried Red Bell Pepper

Photos of the inner surface of the dried red bell pepper were taken with a Nikon D7000 digital camera (Nikon, Tokyo, Japan). A camera lens was mounted above the drought at a height of 100 cm, placed in a black chamber without external light. The material was illuminated with daylight from four fluorescent lamps [24].

The photos of the internal structure were taken with the use of the Skyscan 1272 computer microtomography system (Bruker, Kontich, Belgium). The material was glued with the shorter side to a metal table with a diameter of 25 mm. The scans were performed at a source voltage of 40 kV and current of 193 µA, with a resolution of 25 µm and 0.3° rotation step. The obtained raw images were binarized in the range 25–255, using CTAn software (Bruker) [72]. A photo showing a cross-section in the middle of the material was chosen to illustrate the internal structure.

#### 3.5.2. Chemical Properties of Dried Red Bell Pepper

##### Extract Preparation

A total of 0.3 g of the ground material in the analytical grinder was extracted with 80% ethyl alcohol solution (20 mL) for 20 min, while gently heating it on a heating plate. The solution was filtered through filter paper (84 g/m^2^; Munktell, Germany) and made up to 50 mL with extraction reagent. These extracts were used in the analysis of total phenolic content and antioxidant activity [45].

##### Total Phenolic Content (TPC) of Dried Red Bell Pepper

The color reaction with the Folin–Ciocâlteu reagent (F-C) was used to determine the total content of polyphenols. A total of 0.18 mL of the ethanolic extract, which was diluted with 4.92 mL of distilled water and 0.3 mL of the F-C reagent, was placed in the reaction tube. After 3 min, the reaction was stopped by changing the pH of the solution by adding an anhydrous sodium carbonate solution (170 g/L). The solution was left for one hour at 25 °C, protected from light.

The absorbance was measured by using a UV–VIS spectrophotometer (Evolution 220, Thermo Fisher Scientific, Waltham, MA, USA) at a wavelength of 750 nm [45]. A blank was prepared in the same way; the extract was replaced with an extraction reagent. The analysis was performed in triplicate for each extract. The quantitative content of total polyphenols was presented in relation to the calibration curve made for chlorogenic acid in the range of 0–100 μg/mL. The results were expressed as mg of chlorogenic acid per 100 g of dry substance.

##### Total Carotenoids Content (TCC) of Dried Red Bell Pepper

Total carotenoid content was determined based on the Polish Standard PN-EN 12136: 2000 [73] with modifications. In order to remove interfering macromolecular compounds, e.g., proteins, 0.2 g of ground dried material was diluted with 20 mL of distilled water and mixed with 1 mL of Carrez solution I and 1 mL of Carrez solution II (Carl Roth GmbH, Karlsruhe, Germany). After 4 min of mechanical stirring, the solution was centrifuged. The precipitate was extracted three times in an orbital shaker (Multi Reax Heidolph, Schwabach, Germany) for 4 min, with 20 mL acetone portions, and then centrifuged in a laboratory centrifuge for 7 min at 1000× *g* (Megastar 600R, VWR). The solution collected in the funnel was mixed with 50 mL of petroleum ether with the addition of 10 mL of distilled water. The hydrophobic phase was collected in a centrifuge tube with 1.5 g of anhydrous sodium sulfate and centrifuged. The quantitative content of carotenoids was determined by measuring the absorbance of the ethereal solutions at a wavelength of 450 nm (Spectronic 200; Thermo Fisher Scientific Inc., Waltham, MA, USA). The analysis was conducted in triplicate. Assuming that the extinction coefficient of 1% *β-carotene* in petroleum ether solution is 2592, the dye content in the samples was calculated based on the formula:

(4) $$\mathrm{TCC}\left(mg\;\beta \text{-}\mathrm{carotene}/100g\;\mathrm{dm}\right)=A_{450}\times 10^{5}\times m_{2}/A_{1\mathrm{cm}}^{1\%}\times m_{1}\times \mathrm{dm},$$

where *A*_450_ is the absorbance, *m*_2_ is total extract weight, $A_{1\;\mathrm{cm}}^{1\%}$ is the *β*-*carotene* extinction coefficient in petroleum ether (2592), *m*_1_ is sample weight, and *dm* is dry matter.

##### Antioxidant Activity (DPPH and ABTS Assay) of Dried Red Bell Pepper

The ability to neutralize free radicals was determined by using colored reactions of ethanol extracts of dried pepper with solutions of 1,1-diphenyl-2-picrylhydrazyl radical (DPPH^•^) and the radical cation 2,2-azino-bis(3-ethylbenzothiazoline-6-sulfonate) (ABTS^•+^) [45]. Measurements were made in triplicate. The results are given as the sample concentration necessary to reduce the free radical concentration by 50%.

##### DPPH Assay

The radical solution was prepared 12 h before analysis by dissolving 25 mg of DPPH (Sigma-Aldrich, Steinheim, Germany) in 100 mL of methyl alcohol. Immediately before the analysis, the stock solution was diluted with 80% ethyl alcohol to obtain a mixture whose absorbance measured 515 nm and was in the range of 0.700 ± 0.020. Then, 100, 200, 300, and 400 µL of the sample extract were added to the reaction tubes, supplemented with 80% ethanol to 2 mL and 2 mL of the radical working solution. The mixture was stirred and left without light at room temperature. After 30 min, the absorbance of the colored solutions was measured, and the extinction% of the radical was calculated. On the basis of the obtained results, the curve of the dependence of the substance content on the quench value was plotted. The concentration corresponding to the quenching of 50% of free radicals in the solution was read.

##### ABTS Assay

A stock radical solution was prepared 16 h prior to analysis. Then 38.4 mg of ABTS and 6.6 mg of potassium persulfate were dissolved in 10 mL of distilled water. The radical solution was prepared immediately before the analysis, and a working solution was prepared by diluting the initial mixture about 100 times with 80% ethyl alcohol. The absorbance of the solution prepared in this way, measured at 734 nm, should be within the range of 0.700 ± 0.020. Then, 10, 20, 30, and 40 µL were added to the reaction test tubes, 40 µL of sample extract, and 3 mL each of the radical working solution. After 6 min, the absorbance was measured, and the same was performed as in the previous measurement.

##### Total Sugars Content (TSC) of Dried Red Bell Pepper

The total sugars content (TSC) was determined by using liquid chromatography with an IR detector (Waters, Milford, MA, USA) [74]. Then 0.2 g of the ground material was mixed with 25 mL of Milli-Q ultrapure water at 80 °C and mixed for 4 h on a Multi Reax shaker. The solution was centrifuged, and the supernatant was purified by using an Acrodisc GHP 0.45 µm, 25 mm of diameter syringe filter (Pall Life Sciences, New York, NY, USA). Separation was carried out by using sugar-Pak I cations ion exchange, 10 μm, 6.5 mm × 300 mm analytical column thermostated at 90 °C with a Sugar-PakGuard-Pak insert, 10 µm (Waters, Milford, CT, USA). Separation of 5 µL samples was performed in 20 min with a flow of 0.6 mL min^−1^. Elutions were carried out isocratically, using Milli-Q water as a mobile phase. Quantitative analysis was performed with calibration curves (0–5 mg/mL) for sucrose, D—(+) glucose, and D—(−) fructose standards (Sigma-Aldrich, Steinheim, Germany). The determination was carried out in three repetitions.

##### Thermogravimetry Analysis (TGA)

Thermal stability was determined by using a thermogravimeter (TGA/DSC 3+, Mettler Toledo, Greifensee, Switzerland). The crushed material in the amount of approximately 5 mg was placed in open 70 µL alumina crucibles and subjected to pyrolysis at a temperature of 30 to 600 °C, with a heating rate of 10 k/min, under a nitrogen atmosphere (flow 50 mL/min) [75]. Analysis of the thermograms was performed by using the STAR software (version 16.10) from Mettler Evaluation. Two replications of the analysis were performed.

##### Fourier-Transform Infrared Spectroscopy (FTIR)

Measurements of infrared spectra for dried red pepper were conducted with the use a Cary 630 (Agilent Technologies Inc., Santa Clara, CA, USA) equipped with a single-bounce attenuated total reflectance (ATR) diamond crystal interface. The measurement was carried out in the wavelength range of 650–4000 cm^−1^, at the resolution of 4 cm^−1^, with 32 scans on the spectrum. The analysis was carried out by pressing the dried sample against the crystal with a pressure clamp. Five scans were taken for each sample. All data were recorded by MicroLab FTIR software [76].

### 3.6. Statistical Analysis

The dried red bell pepper samples were subjected to the ANOVA analysis with the use of TIBCO company software (STATISTICA program, version 13, Palo Alto, CA, USA). The samples were divided into homogenous groups with the use of Tukey’s test (α = 0.05). In some cases, the data were subjected for Pearson’s correlation analysis to determine the relationship between selected parameters. Furthermore, based on the determined parameters, a Cluster Analysis (using Ward method for grouping) was carried out in order to comprehensively evaluate the obtained results and group the samples.

## 4. Conclusions

The convective and microwave–convective drying allowed us to obtain dried material with water activity much below 0.6. The use of microwave power reduced the drying time by 76% in comparison to hot-air-drying, while the application of pretreatment further shortened the drying time for both convective and microwave–convective drying. The shortest drying time was obtained for the blanched sample; however, in this case, vitamin C was significantly reduced.

The samples that were dried with the microwave–convective method were characterized by higher porosity and better rehydration properties, especially when the non-thermal processes, such as ultrasound (US) and pulsed electric field (PEF), were used. This was also confirmed by the images obtained from microtomography. The changes of color of the convective dried samples were more intensive than for the samples dried with the use of microwaves, while the non-thermal processing resulted in higher values of total color difference in comparison to thermal treatment. Bioactive compounds, such as vitamin C and total polyphenols content, in dried red bell pepper subjected to non-thermal processing were on the same or lower level as untreated dried material. This was probably linked with a long period of drying via hot-air-drying or as a result of uneven heating and the possibility of overheating the samples during microwave-drying. Meanwhile, total carotenoid content after non-thermal processing and drying was characterized usually by a higher amount of these compounds. Taking into account the total antioxidant activity of the samples dried with the use of microwave, it was seen that they had higher antioxidant activity. It is noteworthy that the PEF-treated samples before drying were characterized by higher antioxidant activity when compared to untreated, blanched, and sonicated dried materials. After we carried out both thermal and non-thermal pretreatments in water, the red bell pepper contained lower sugar content, in the range of 12.7% to 31.3%, in comparison to untreated dried material. This might be beneficial for obtaining food with lower sugar content to reduce the calorific value of the product.

Generally, the method of drying, as well as the method of pretreatment, has a strong effect on the final product. However, thermal processing before drying differs the samples in comparison to non-thermal processing. Furthermore, even the sonication and pulsed electric field cause a similar effect as cell-wall rupturing, and structure changes, the mechanism is different, and this resulted in different properties for the samples, as was confirmed by PCA analysis.

## Figures and Tables

**Table 1:** Convective (CV) and microwave–convective (MV) drying time of red bell pepper (to moisture ratio MR = 0.02). Data are presented as a mean (from 2 repetitions) ± standard deviation. The different lowercase letters in row show different homogeneous groups, and different capital letters in columns show different homogeneous groups (Tukey’s HSD, α = 0.05).

Drying Time to MR = 0.02 (min)					
Drying Method	Untreated	BL-W	US	PEF1	PEF3
CV	810 ± 65 aA	332 ± 45 cA	666 ± 45 bA	554 ± 30 bA	526 ± 45 bA
MV	190 ± 22 aB	129 ± 19 bB	152 ± 16 abB	153 ± 16 abB	147 ± 28 abB

**Table 2:** Water activity (a_w_), porosity, and rehydration rate of convective (CV) and microwave–convective (MV) dried red bell pepper subjected to thermal or non-thermal treatment prior to drying. Data are presented as a mean (from 3 repetitions) ± standard deviation. The different letters in columns show different homogeneous groups (Tukey’s HSD, α = 0.05).

Dried Red Bell Pepper	a_w_ (−)	Porosity (%)	RR (−)
Convective Dried Red Bell Pepper			
CV_Untr	0.314 ± 0.004 ef	4.7 ± 0.7 a	2.64 ± 0.05 a
CV_BL-W	0.270 ± 0.005 d	13.6 ± 1.3 bc	4.21 ± 0.01 cd
CV_US	0.227 ± 0.005 b	10.8 ± 0.9 b	2.60 ± 0.19 a
CV_PEF1	0.258 ± 0.006 cd	10.4 ± 0.4 b	3.00 ± 0.08 ab
CV_PEF3	0.201 ± 0.004 a	16.4 ± 0.9 c	2.87 ± 0.08 a
Microwave–convective dried red bell pepper			
MV_Untr	0.393 ± 0.005 g	10.0 ± 0.9 ab	3.88 ± 0.33 bcd
MV_BL-W	0.242 ± 0.005 bc	10.5 ± 1.8 ab	4.06 ± 0.17 cd
MV_US	0.234 ± 0.006 b	39.9 ± 1.8 e	4.68 ± 0.29 d
MV_PEF1	0.318 ± 0.010 f	25.7 ± 1.8 d	4.18 ± 0.44 cd
MV_PEF3	0.294 ± 0.007 e	26.4 ± 1.9 d	3.47 ± 0.20 abc

**Table 3:** Parameters of treatment of red bell pepper before subjecting to drying.

Treatment	Description and Treatment Parameters		
Thermal treatment			
UNTR	Untreated	-	
BL-W	Blanching in water	98 °C, 3 min	
Non-thermal treatment			
US	Ultrasound	Sonication time, 30 min; ultrasonic bath with frequency of 21 kHz; ultrasound intensity equal to 3 W/cm^2^	
PEF1	Pulsed electric field	Electric field intensity of 1.07 kV/cm	Specific energy intake: 1.0 kJ/kg
PEF3	Pulsed electric field		Specific energy intake: 3.0 kJ/kg

## Data Availability

The data presented in this study are available on request from the corresponding author.

## Sample Availability

Samples are not available from the authors.
